# Evaluation of wastewater SARS-CoV-2 surveillance for monitoring COVID-19 trends and early warning in Sweden, 2023–2024

**DOI:** 10.1371/journal.pone.0358326

**Published:** 2026-09-21

**Authors:** Anna Ohlson, Moa Rehn, Sharon Kühlmann Berenzon, Erik Sturegård, Rakel Brodin, Anna J. Székely, Sofia Persson, Ilias Galanis

**Affiliations:** 1 Department of Communicable Disease Control and Health Protection, Public Health Agency of Sweden, Solna, Sweden; 2 ECDC Fellowship Programme, Field Epidemiology path (EPIET), European Centre for Disease Prevention and Control (ECDC), Stockholm, Sweden; 3 Department of Public Health Analysis and Data Management, Public Health Agency of Sweden, Solna, Sweden; 4 Department of Aquatic Sciences and Assessment, Science for Life Laboratory, Swedish University of Agricultural Sciences, Uppsala, Sweden; SKUMS: Shahrekord University of Medical Science, IRAN, ISLAMIC REPUBLIC OF

## Abstract

Wastewater surveillance (WWS) of SARS-CoV-2 RNA levels offers a way to monitor virus circulation independently of clinical testing. With COVID-19 now endemic, clarifying the role of WWS within routine surveillance is essential. The aim was to evaluate the potential of WWS as an early warning indicator for COVID-19 in Sweden, based on its correlation with reported case data. The study includes data from July 2023 to December 2024, from 19 wastewater treatment plants (WWTPs), covering 43% of Sweden’s population, and surveillance data of reported COVID-19 cases. Analyses were performed by WWTP, separately for Stockholm (4 WWTPs) and at a national level (19 WWTPs). We compared wastewater SARS-CoV-2 levels to reported COVID-19 cases using cross-correlation and prediction metrics following trend classification. Correlations and classification metrics were highest without time shifts. SARS-CoV-2 levels in wastewater were strongly correlated with reported COVID-19 cases, with median cross-correlation values of 0.80 across all WWTPs and 0.87 at Stockholm and national levels. Positive predictive values of wastewater data to reported cases ranged from 63–74%, and negative predictive values from 85–95%. Sensitivity and specificity in Stockholm reached 78% and 87%; at national level, 72% and 84%, respectively. Under the evaluated weekly sampling and reporting system, WWS did not demonstrate a consistent early-warning advantage over reported cases but may support monitoring of trends when clinical testing is reduced.

## Introduction

Following the acute phase of the COVID-19 pandemic, SARS-CoV-2 continues to circulate in Sweden, although clinical testing has declined substantially compared with earlier pandemic phases. While routine surveillance continues to rely primarily on case-based surveillance and other established indicators, wastewater surveillance (WWS) has emerged as a complementary approach that may provide additional information on community transmission. Wastewater surveillance (WWS) for SARS-CoV-2 is based on detecting viral RNA shed through feces, urine, and respiratory secretions [1,2]. Studies estimate that approximately half of infected individuals shed SARS-CoV-2 RNA in feces, including both symptomatic and asymptomatic cases [1,3,4]. Consequently, WWS could offer population-level monitoring without relying on clinical testing and potentially enabling the early detection of changes in community transmission before becoming apparent in case-based surveillance systems.

During the COVID-19 pandemic, WWS rapidly gained international attention as a complementary tool for tracking SARS-CoV-2 circulation and monitoring variant emergence and spread [5]. Following recommendations from the European Commission in early 2021, multiple European Union member states established WWS programs to systematically track SARS-CoV-2 levels and variants of concern [6–9].

Studies conducted during the early days of the pandemic and during the Delta variant period, reported that wastewater signals preceded reported clinical cases by approximately one week [5,10,11]. Studies conducted during the Omicron period, both in settings with extensive clinical testing, such as Denmark [9], and in those with more limited testing, including Belgium, Italy, and Scotland [12–14], found strong correlations between wastewater data and reported cases, but did not demonstrate significant early-warning advantages compared to reported cases. A study from Norway suggested early-warning potential during Omicron; however, short-term fluctuations complicated interpretation of the signals [15].

In Sweden, surveillance of COVID-19 relies on multiple indicators, including mandatory reporting of laboratory-confirmed cases, hospitalizations and intensive care admissions, and mortality data [16]. Since April 2020, wastewater-based SARS-CoV-2 data have been regularly published by research groups, notably by the Swedish University of Agricultural Sciences (SLU), which provides open-access weekly data [17]. However, despite the availability of these data, the added value of WWS within the Swedish surveillance system, particularly its ability to provide early warning of increasing transmission, has not been comprehensively evaluated. While many correlation studies exist from early phases of the pandemic, few have assessed WWS as a leading indicator under late-pandemic/Omicron-dominant conditions characterized by reduced testing and a markedly changed care-seeking behavior. Therefore, in this study we addressed the real time utility and added value of WWS under endemic-like conditions using multi analytical approach.

The aim of this study was to evaluate the correlation between SARS-CoV-2 levels in wastewater and reported COVID-19 cases in Sweden, and assess the potential of wastewater surveillance as an early warning indicator within the national COVID-19 surveillance system.

## Methods

### Study period

This study included data on SARS-CoV-2 levels in wastewater and reported COVID-19 cases in Sweden over a 72-week period, from 24 July 2023 (week 30) to 15 December 2024 (week 50). Throughout this period, the Omicron variant of SARS-CoV-2 was circulating [18]. Clinical testing recommendations remained unchanged throughout the study period, prioritizing patients in healthcare and individuals in elderly care homes who were at increased risk of severe illness [19]. The 72-week period was chosen because it represents the full phase during which all 19 wastewater treatment plants (WWTPs) sampled in Sweden participated consistently and Omicron variants dominated circulation. These 19 WWTPs constituted the entire national WWTP sampling network in Sweden, selected to ensure broad geographical coverage and to include the largest population centers.

### Data collection

#### Current surveillance indicator - COVID-19 reported cases.

In Sweden, COVID-19 is a notifiable disease under the Communicable Diseases Act (2004:168), requiring laboratories to report all positive PCR and antigen tests, and medical doctors to report all positive bedside antigen tests to the Public Health Agency of Sweden (PHAS) through Sminet, the Swedish national surveillance system for notifiable communicable diseases. According to the PHAS surveillance protocol, an individual is considered a new COVID-19 case if at least six months have passed since their last reported infection. For this study, all reported COVID-19 cases registered in SmiNet between 24 July 2023 and 15 December 2024 were included. The data were accessed on 3 March 2025. Individual notifications were aggregated into weekly regional and national case counts based on notification date. COVID-19 surveillance data were accessed as part of routine national public health surveillance activities at the Public Health Agency of Sweden. Only aggregated data are presented in the manuscript, and no individual-level personal data were used in the analyses.

#### Alternative surveillance indicator – Wastewater SARS-CoV-2 levels.

**Wastewater sampling:** Wastewater sampling, processing and reverse transcription quantitative polymerase chain reaction (RT-qPCR) analyses were performed by SLU. During the study period, wastewater samples were collected weekly from 19 WWTPs distributed across 14 out of Sweden’s 21 regions. The WWTPs covered between 4 500 and 800 000 inhabitants in their catchment areas, corresponding to 1–52% of the regional population (Supporting information; S4 Fig in the S4 File, S2 Table in the S2 File). In Stockholm, four WWTPs covered 86% of the Stockholm region population. The included 19 WWTPs covered 43% of Sweden’s total population. While the included WWTPs did not cover the entire Stockholm region or Sweden, aggregated wastewater indicators were calculated for Stockholm and across all participating WWTPs in Sweden. These are referred to as the Stockholm and national wastewater indicators throughout the manuscript.

Eighteen plants collected one 24-hour flow-proportional composite sample every Monday. One plant (Uppsala) collected daily 24-hour composite samples (Monday–Sunday); these daily samples were stored refrigerated at 4 °C and subsequently aggregated by SLU into weekly flow-proportional composites. At Stockholm-Bromma, Stockholm-Henriksdal, and Uppsala, samples from multiple inlets were flow-proportionally mixed into a single sample, as previously described [20]. Samples were transported on cold packs, and kept at 4 °C until analysis which was typically carried out within 48 hours from collection.

**Nucleic acid extraction:** Total nucleic acids were extracted from raw wastewater using the Maxwell® RSC Enviro TNA kit (Promega, AS1831) on the Maxwell® RSC instrument (AS4500) with the PureFood GMO and Authentication program, as previously described [21]. Each sample was split into two subsamples; 40 ml of each was processed and eluted in 100 µl of nuclease-free water. A negative control (40 ml tap or Milli-Q water) was included in each extraction round. Extracts were stored at −80 °C until RT-qPCR the following day.

**RT-qPCR analysis:** Viral genomic copy numbers were estimated by RT-qPCR using a Bio-Rad CFX Duet or CFX96 system. Reactions were prepared with the Reliance One-Step Multiplex Supermix (Bio-Rad, 12010221) supplemented with 1 mg/ml bovine serum albumin (Thermo Fisher, AM2618). Each reaction consisted of 15 µl master mix and 5.0 µl RNA template.

SARS-CoV-2 was detected using primer and probe sequences from the CDC Influenza SARS-CoV-2 Multiplex Assay [22], with final concentrations of 250 nM of forward, reverse primer, and probe (S1 File). Pepper Mild Mottle Virus (PMMoV) was included as a process control and population normalization marker, given its consistent high abundance in Swedish wastewater [23], using previously published primer and probe sequences [24,25], at final concentrations of 600 nM forward primer, 800 nM reverse primer, and 200 nM probe. Beginning in week 12 of 2024, an internal amplification control (IAC) was run in duplex with the PMMoV assay to assess RT-qPCR inhibition, using previously described primer, probe, and target sequences [26] at 400 nM forward primer, 400 nM reverse primer, 200 nM probe, and 10^4^ copies of synthetic RNA template per reaction.

The thermal cycling conditions were as follows: 50 °C for 30 min, 95 °C for 5 min, followed by 45 cycles of 95 °C for 15 s and 55 °C (SARS-CoV-2) or 58 °C (PMMoV/IAC) for 30 s.

Viral genome copies were quantified using standard curves generated from serial tenfold dilutions of double-stranded DNA gBlock fragments (Integrated DNA Technologies, IDT) containing the target amplicon regions for SARS-CoV-2 and PMMoV. Primers, probes, and synthetic RNA template were also purchased from IDT.

Two technical replicates were analyzed per subsample, and each plate contained two no-template controls. RT-qPCR results were first averaged across replicate wells and then across the two subsamples to obtain the final value. SARS-CoV-2 concentrations were normalized to PMMoV by dividing the SARS-CoV-2 copy number by the corresponding PMMoV copy number and multiplying by 1,000 (copies per 1,000 PMMoV copies).

**Quality control:** The 95% limit of detection (LOD95%) and the limit of quantification (LOQ) were determined using serial dilution series of gBlock standards. For SARS-CoV-2, these were 3.14 and 3.52 copies per reaction, respectively. Concentrations falling below the LOQ but above the LOD95% were imputed as the midpoint between the LOD95% and LOQ, calculated as (LOD95% + LOQ)/ 2.

RT-qPCR inhibition was evaluated using the IAC. Inhibition was calculated as the difference between the IAC quantification cycle (Cq) value in each wastewater sample well and the mean IAC Cq value in non-template controls (NTCs). A Cq shift greater than 2 cycles was considered indicative of unacceptable inhibition. Extraction variability was assessed from duplicate RNA extractions; samples with a relative standard deviation (RSD) exceeding 100% between duplicates were excluded from further analysis.

### Data analysis

#### Handling missing data and aggregation of wastewater levels.

Of the 1,368 expected WWTP weekly samples (19 sites × 72 weeks), 97 (7%) were missing. Data from the WWTP in the Jönköping region were unavailable from week 19 to week 43 in 2024 (n = 25), while the remaining missing samples (n = 72) occurred sporadically across 17 WWTPs. Single missing values were imputed using the average of the preceding and following week. After imputation, 22 values remained missing across eight WWTPs (1.6% of all data), reflecting periods of two or more consecutive missing wastewater measurements. These weeks (and corresponding case data) were excluded from the correlation analyses. This simple interpolation was used because cross-correlation analyses require complete time series, and adjacent-week averaging was considered minimally intrusive and trend-preserving.

For Stockholm (4 WWTPs) and national level (19 WWTPs), weighted averages of SARS-CoV-2 levels in wastewater were calculated with weights based on population sizes within the respective WWTPs catchment areas. Four weeks where a WWTP had missing data, that WWTP was excluded from both the calculation and the population weight.

#### Cross-correlation analysis.

To assess how well weekly SARS-CoV-2 levels in wastewater reflected reported cases, we performed cross-correlation analyses across multiple time lags using the Pearson correlation coefficient. For interpretation, we focused on lags of 0 to ±3 weeks by shifting wastewater data ahead of and behind reported cases. We also stratified the analysis by periods of rising case numbers (weeks 39–45, 2023) and declining case numbers (week 50, 2023, to week 4, 2024), applying the same 0 to ±3-week time shifts. The correlation coefficient *r* was calculated between the two indicators for each WWTP, Stockholm and national level. We reported *r* and summarized the results across all WWTPs using the median *r* and interquartile range (IQR). To evaluate whether WWTP population coverage influenced the results, we examined the relationship between *r* and the proportion of the regional population within WWTP catchment area using Spearman’s rank correlation.

#### Trend estimation and weekly changes.

Three-week trends were estimated using Poisson regression models for reported COVID-19 case data, with population size as an offset variable, and linear regression models on log10-transformed wastewater data for viral levels. The percent weekly changes (PWC) were then estimated from the slope as: PWC = (10slope–1)*100 [25].

We calculated changes in reported cases and wastewater virus levels using (1) 1-week percent change and (2) PWC. Changes in reported cases and wastewater data were classified as ‘increases’ using two thresholds (>10% and >25% [27]) or ‘no increase’.

#### Predictive values, sensitivity and specificity.

We calculated prediction metrics using reported COVID-19 cases as the reference surveillance indicator. Weeks in which both reported cases and wastewater SARS-CoV-2 levels exceeded the predefined threshold were classified as true positives, whereas weeks in which only wastewater exceeded the threshold were classified as false positives relative to the reference indicator. Similarly, weeks in which only reported cases exceeded the threshold were classified as false negatives, and weeks in which neither indicator exceeded the threshold were classified as true negatives. These metrics were assessed with and without shifting WWS data 1–3 weeks forward, for Stockholm and national levels. Weeks with data below the 20th percentile were removed from the calculation of positive/negative predictive values (PPV/NPV), sensitivity and specificity to minimize noise that disproportionately affects the percent changes.

Weeks in which both reported cases and wastewater SARS-CoV-2 levels exceeded the predefined threshold were classified as true positives, whereas weeks in which only wastewater exceeded the threshold were classified as false positives relative to the reference indicator. Similarly, weeks in which only reported cases exceeded the threshold were classified as false negatives, and weeks in which neither indicator exceeded the threshold were classified as true negatives.

All statistical analyses were performed using R version 4.2.0 (www.r-project.org).

### Ethics statement

This study reports aggregated data on reported COVID-19 cases and SARS-CoV-2 levels in wastewater. COVID-19 case data were collected as part of the Public Health Agency of Sweden’s mandate for infectious disease surveillance and control as defined by national legislation. As the study was based solely on aggregated surveillance data, ethical approval was not sought.

## Results

**Fig 1:**
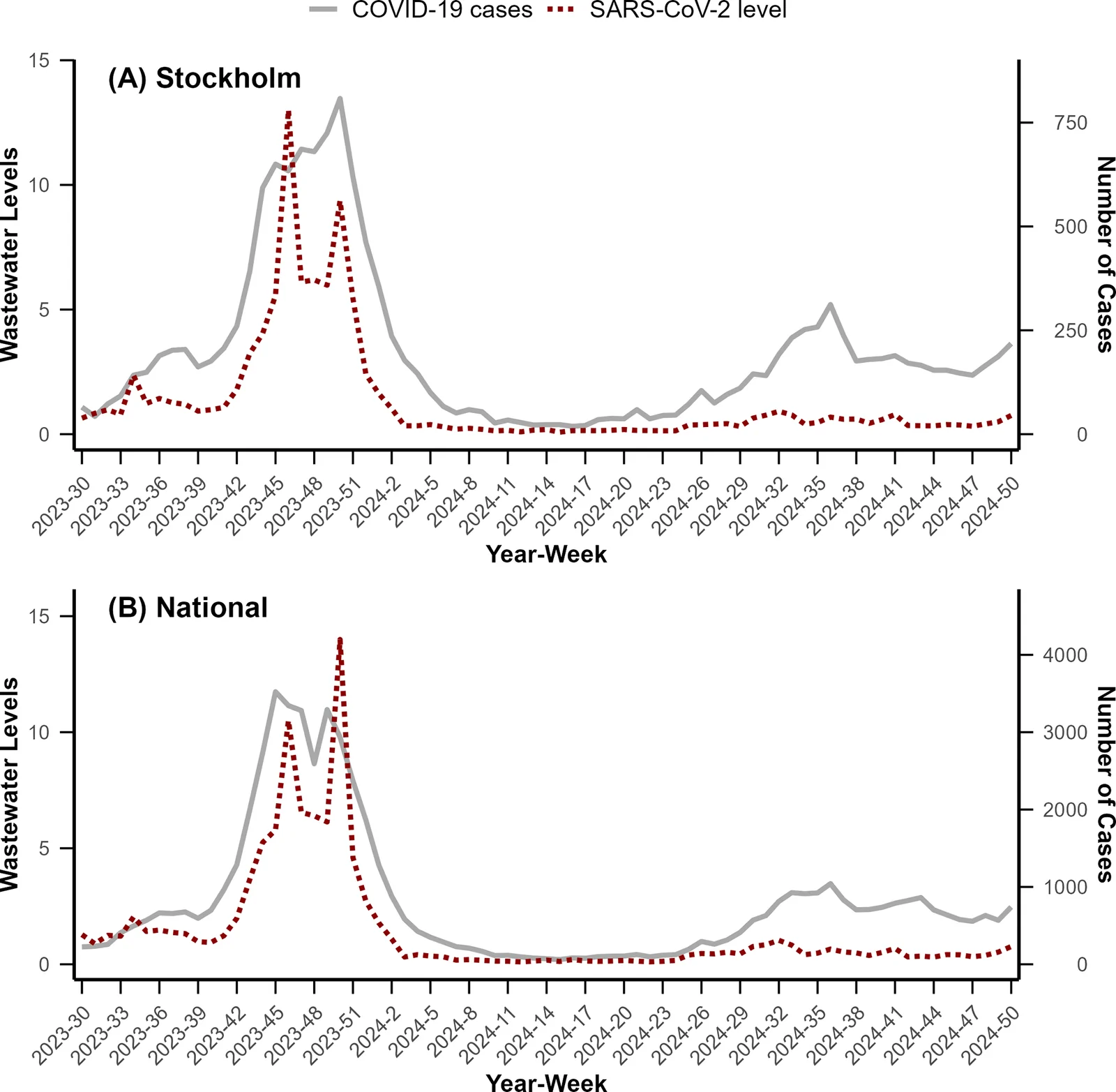
Weekly COVID-19 cases and wastewater SARS-CoV-2 levels in Stockholm and Sweden. Weekly reported COVID-19 cases (grey line) and SARS-CoV-2 RNA levels in wastewater (dotted red line) in (A) Stockholm and (B) National level. Wastewater SARS-CoV-2 RNA levels were population-weighted and expressed as SARS-CoV-2 copies per 1,000 PMMoV copies.

Fig 1A and 1B illustrate SARS-CoV-2 levels in wastewater alongside reported COVID-19 cases for Stockholm and the national level. Two case surges were observed: the first in autumn–winter 2023 with pronounced peaks in both indicators, and the second in late summer–autumn 2024, with lower magnitudes.

### Cross-correlation analysis

**Fig 2:**
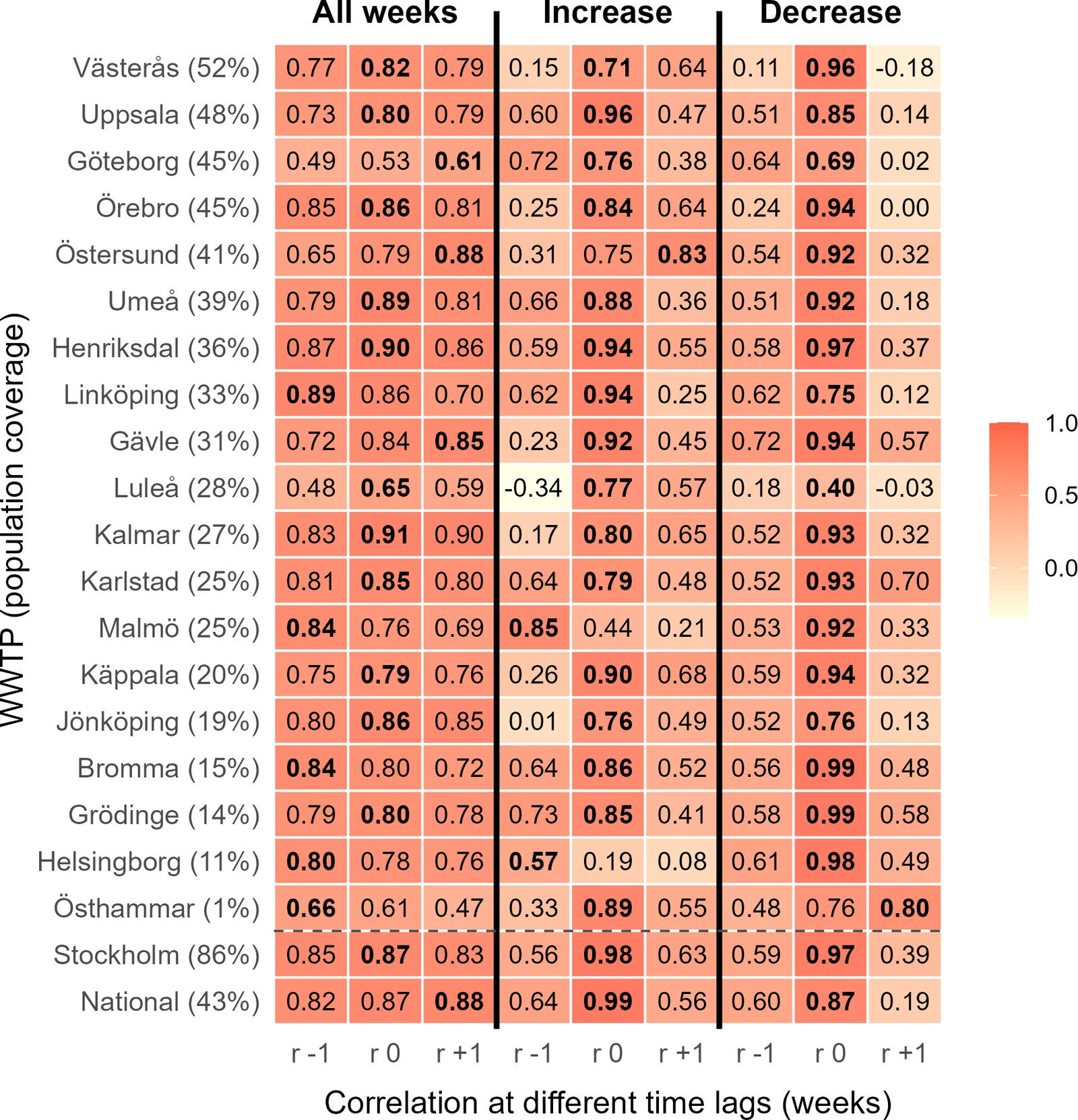
Cross-correlation between wastewater SARS-CoV-2 levels and COVID-19 cases. Correlations are shown for all weeks (n = 72), weeks of increasing COVID-19 cases (n = 7), and weeks of decreasing COVID-19 cases (n = 7). Correlation coefficients (r) are shown at lag −1 to +1, where negative lags indicate wastewater signals preceding reported cases and positive lags indicate wastewater signals lagging behind cases. Results are presented for 19 wastewater treatment plants (WWTPs), Stockholm (4 WWTPs), and the national level (19 WWTPs). The highest correlation for each WWTP is shown in bold.

The strongest correlations (r) were seen at no time shift across 11 WWTPs and the Stockholm level, with WWS one week ahead of cases for 5 WWTPs, and one week behind for 3 WWTPs and the national level (Figs 2 and S2 in S5 File). At no time shift, the median *r* across the 19 WWTPs was *r* = 0.80 (IQR: 0.78–0.86), with *r* = 0.87 for both Stockholm and national level. When WWS led by one week, the median *r* was 0.79 (IQR: 0.73–0.83), with *r* = 0.85 for Stockholm and *r* = 0.82 nationally. When WWS lagged by one week, the median *r* across WWTPs was 0.79 (IQR: 0.71–0.83), with *r* = 0.83 for Stockholm and *r* = 0.88 nationally. No significant correlation was found between *r* at no time shift and WWTP population coverage at the regional level (ρ = 0.22, p-value = 0.36).

During the seven-week period of increasing cases, the strongest *r* values were observed with no time shift across 17 WWTPs, as well as at the Stockholm and national levels (Figs 2, S3 in S5 File). The median *r* at no shift was 0.84 (IQR: 0.76–0.90) across WWTPs, with *r* = 0.98 for Stockholm and *r* = 0.99 nationally. Correlations declined notably when WWS led by one week: median *r* = 0.57 (IQR: 0.24–0.64), with *r* = 0.56 for Stockholm and *r* = 0.64 nationally. Similarly, during the seven-week decline period, the highest *r* values were at no time shift for 18 WWTPs: median *r* = 0.93 (IQR: 0.81–0.95), with *r* = 0.97 for Stockholm and *r* = 0.87 nationally (Figs 2 and S4 in S5 File). Correlations dropped when WWS led by one week: median *r* = 0.53 (IQR: 0.51–0.59), with *r* = 0.59 for Stockholm and *r* = 0.60 nationally.

### Predictive values, sensitivity and specificity

For the week-to-week analysis, the highest PPVs were observed at >10% threshold with no shift in time, reaching 63% at both Stockholm and national level, meaning that an increase in wastewater corresponded to an increase in reported cases in about 6 out of 10 occasions. At >25%, PPVs fell below 50%, meaning that less than half of the weeks with increase in wastewater had a corresponding increase in reported cases. NPVs were overall high, with the highest values at >25% threshold with no time shift (84% Stockholm, 85% national), meaning that when there was no increase in wastewater, there was also no increase in reported cases in 8 out of 10 occasions. For the 3-week trend analysis, PPVs peaked at >10% threshold with no time shift (74% Stockholm, 68% national) and NPVs peaked at >25% threshold with no time shift (95% Stockholm, 90% national).

**Fig 3:**
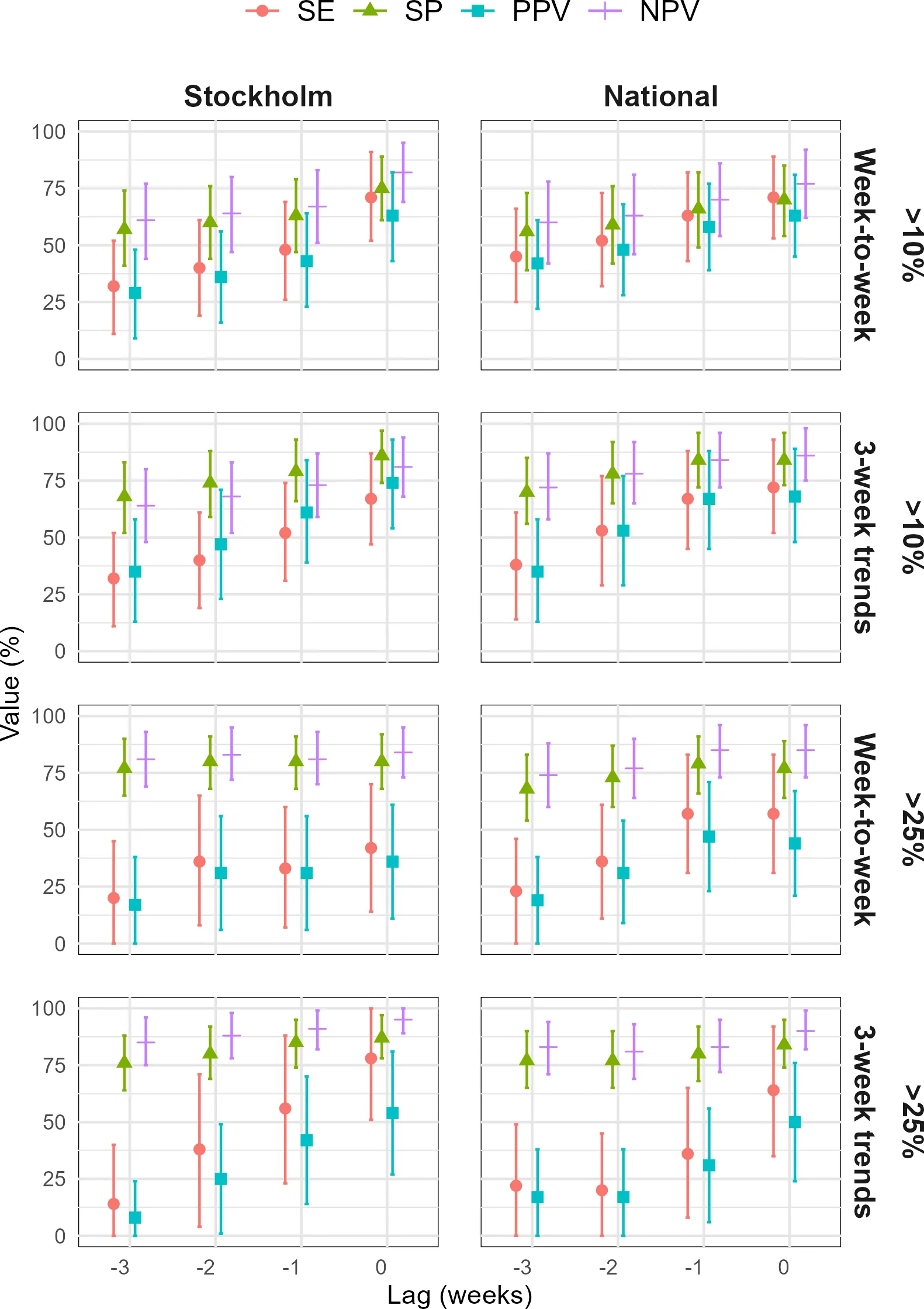
Performance metrics for wastewater SARS-CoV-2 surveillance. Sensitivity (SE), specificity (SP), positive predictive value (PPV), and negative predictive value (NPV) with 95% confidence intervals for wastewater SARS-CoV-2 levels compared with reported COVID-19 cases. Metrics are shown for two thresholds of increase (>10% and >25%) and for week-to-week changes and 3-week trends. Analyses included lag periods where wastewater data were shifted 0 to 3 weeks ahead of reported cases.

Sensitivity and specificity were also highest overall at no time shift across both week-to-week and 3-week trends, with the highest values generally seen for the 3-week trend. For Stockholm, the highest values were for the 3-week trend at no time shift and >25% threshold, with 78% sensitivity and 87% specificity. That is, 78% of case increases coincided with wastewater increases, and 87% of stable weeks showed no increase in wastewater. For the national level, the highest values were for the 3-week trend at no time shift and >10% threshold, with 72% sensitivity and 84% specificity. See Fig 3 for detailed results and S2 Table in S3 File for the numbers of true positives, false positives, true negatives, and false negatives underlying the classification metrics.

## Discussion

This study evaluated the correlation between wastewater surveillance (WWS) and reported COVID-19 cases in Sweden, and assessed the potential of WWS as an early warning indicator. Our results show that WWS closely correlate with reported cases but do not provide earlier signals of increase in reporting.

Several methodological factors may affect how well wastewater signals reflect reported COVID-19 cases, including flow patterns, dilution from rain or snowmelt, sewer travel time, and analytical variation. Similar sources of noise were described by van Boven et al. [28], highlighting substantial day-to-day variation in wastewater measurements partly driven by wastewater network characteristics. Such characteristics (e.g., catchment size, hydraulic residence time, infiltration/inflow and stormwater contributions) differ between sites and may influence observed SARS-CoV-2 RNA levels. We accounted for much of this variability by normalising SARS-CoV-2 concentrations to PMMoV, which helps adjust for fluctuations in dilution and flow (including snowmelt-related increases in northern sites such as Umeå and Östersund). However, PMMoV may not fully capture all site-specific sewer system effects.

In our study, correlations between wastewater SARS-CoV-2 levels and reported cases were generally moderate to strong across WWTPs (typically r ≈ 0.7–0.9 at lag 0). Cross-correlations were strongest for most WWTP when wastewater and case time series were temporally aligned (lag 0), while shifting the wastewater signal by ±1 week generally reduced correlations—most notably during periods of increasing or decreasing case incidence. This suggests that wastewater surveillance primarily reflects, rather than consistently anticipates, trends in reported cases, regardless of WWTP population coverage. Similar findings have been reported in other Omicron-period studies [9,12–14]. The lack of a consistent leading pattern may relate to biological characteristics of Omicron infections. Also, a single weekly sample limits temporal precision, higher-frequency sampling might improve alignment between case and wastewater signals.

In this analysis, the PPV refers to the proportion of weeks where an increase in wastewater levels of SARS-CoV-2 was aligned with an increase in reported COVID-19 cases, based on predefined thresholds. PPVs were moderate overall, raising concerns about epidemiological interpretation of WWS in real time and a key challenge for its operational use. The WWS signals not reflected in reported cases may reflect transmission that are undetected in case-based surveillance, or be temporary fluctuations due to for instance changing environmental conditions, shedding variation, or measurement artifacts. This aligns with findings from Norway [15]. Because reported COVID-19 cases were used as the reference surveillance indicator rather than a complete measure of community transmission, PPV, NPV, sensitivity, and specificity should be interpreted accordingly.

NPVs were consistently high, meaning that when wastewater signals did not show an increase, as defined by our thresholds, there was usually no increase in reported cases. This suggests that WWS may be more reliable for confirming periods of low or stable case trends. Specificity was overall high, meaning that WWS accurately detected stable case trends, whereas sensitivity, the ability to identify increase in cases, was generally lower than specificity. All metrics – PPV, NPV, sensitivity, and specificity – were strongest when WWS and reported cases were aligned in time, showing that WWS mirrored the trends in reported cases better than providing an earlier signal. Wide confidence intervals due to few weeks with clear increase limit certainty and warrant cautious interpretation. In particular, there were few weeks in our data with increases above 25%, and a longer observation period would be needed to properly evaluate this.

Using a combination of analytical approaches provided a broader understanding of the potential utility of WWS. Cross-correlation confirmed overall alignment with reported cases. Classification metrics helped assess whether weekly signals in wastewater corresponded with signals based on reported cases. The results showed some potential, especially for identifying weeks without increase in cases, that is, when wastewater did not show an increase, most often cases did not increase either. This suggests that wastewater surveillance may be used particularly for confirming stable case levels.

A limitation of this study is the discrepancy between the populations represented in wastewater data (WWTP catchment areas) and case data (covering all residents in each region). This is particularly relevant at the national level, where not all regions had a WWTP included, but all contributed case data. This was intentional, as we aimed to assess WWS as a regional and national epidemiological indicator. The difference in population coverage may have led to an underestimation of the correlation between the two indicators, especially in regions with limited WWTP coverage. Still, our results were directionally consistent across regions despite catchment variations (1–52%), as well as in Stockholm (86%) and nationally (43%). Furthermore, no significant association was observed between WWTP population coverage and the correlation coefficient across regions, suggesting that differences in catchment coverage did not systematically influence the agreement between wastewater and reported case data. In addition, the cross-correlation analyses may have been influenced by autocorrelation and shared temporal trends in the two surveillance indicators, particularly during the short analyses of increasing and decreasing case numbers. Consequently, the reported correlation coefficients should be interpreted as descriptive measures of temporal agreement rather than evidence of predictive relationships.

Reported cases reflect individuals who seek care and are tested, whereas WWS estimates viral shedding from all infected individuals in the catchment area. Due to limited data on shedding-to-reporting delays, we assumed both indicators reflect the same week, possibly causing some temporal misalignment. In our data, for 18 out of 19 WWTPs, the WWS represents a 24-hour snapshot of viral levels in the WWTP (Monday), while case data covers the detection of cases in healthcare during a full week (Monday–Sunday). Consequently, a lead of only a few days may have appeared as a zero-week lag in our weekly analyses. However, in one WWTP (Uppsala) – where daily samples were pooled into weekly composites – the correlation were consistent with the other 18 WWTPs. Improvements in estimating population-level viral shedding could further enhance data stability and correlations with reported cases. For instance, quantifying viral genomic copy numbers using digital PCR (dPCR) rather than qPCR may reduce analytical variability, and alternative normalization methods could improve alignment with clinical case trends [29–32].

While our evaluation suggests that WWS, as currently sampled and analyzed, has limited value as an early warning signal for COVID-19 epidemics in real-time surveillance during the endemic phase in Sweden, it could be valuable in other contexts. Previous studies have shown that WWS can help detect and monitor the emergence of new SARS-CoV-2 variants [6,8]. In scenarios where clinical testing is unavailable or unreliable, WWS could play an important role to monitor COVID-19 trends.

## Conclusion

Quantitative WWS in Sweden during the Omicron-dominated study period effectively tracked COVID-19 trends but did not provide a consistent weekly-scale early-warning advantage over reported COVID-19 cases under the current weekly sampling, laboratory and reporting procedures. Temporary fluctuations in wastewater levels from single sampling occasions can make short-term interpretation difficult. Future work should focus on improving sampling strategies and quantification methods to reduce variability and enhance data stability.

## Supporting information

S1 File Supporting methods: RT-qPCR assay details. (PDF)

S2 File Summary of included wastewater treatment plants (WWTPs), catchment population coverage, and regional population coverage. (PDF)

S3 File Summary of the classification analysis. (DOCX)

S4 File Geographical location of included Swedish regions and wastewater treatment plants. (PDF)

S5 File Cross-correlation results between wastewater SARS-CoV-2 levels and reported COVID-19 cases for weekly data, periods of increasing COVID-19 case numbers and periods of decreasing COVID-19 case numbers. (PDF)

S6 File Supporting data; aggregated weekly wastewater SARS-CoV-2 levels, reported COVID-19 cases, cross-correlation results, and prediction model outputs underlying the analyses and figures. (XLSX)
