# Supplementary material for: Evaluation of wastewater SARS-CoV-2 surveillance for monitoring COVID-19 trends and early warning in Sweden, 2023–2024
Source: PLoS One. 2026 Sep 21;21(9):e0358326. doi: 10.1371/journal.pone.0358326 (PMC13592587; doi:10.1371/journal.pone.0358326)
Supplement: Supplementary file 1 [file pone.0358326.s001.pdf]

S1 File. Supporting information methods – RT-qPCR assay details

This supporting material is supporting information alongside the article *"Evaluation of wastewater SARS-CoV-2 quantification as a surveillance indicator for COVID-19 in Sweden, 2023–2024"*.

SARS-CoV-2 primers and probe

SARS-CoV-2 was analyzed using the primers and probe from the CDC Influenza SARS-CoV-2 Multiplex Assay [1];

- **SC2-F (Forward primer):** 5′-CTG CAG ATT TGG ATG ATT TCT CC-3′
- **SC2-R (Reverse primer):** 5′-CCT TGT GTG GTC TGC ATG AGT TTA G-3′
- **SC2-P (Probe):** 5′-/TexRd-XN/ATT GCA ACA/TAO/ATC CAT GAG CAG TGC TGA CTC/3IAbRQSp/-3′

Fluorophore and quencher details

TexRd = Texas Red; TAO = internal quencher; 3IAbRQSp = 3′ Iowa Black quencher.

Reference

1. Centers for Disease Control and Prevention. *CDC Influenza SARS-CoV-2 (Flu SC2) Multiplex Assay*. [Internet]. Atlanta, GA: U.S. Department of Health & Human Services; [updated 2023 Nov 9]; Available from: https://www.cdc.gov/flu/php/laboratories/influenza-sars-cov-2-multiplex-assay.html
