# Supplementary material for: Evaluation of wastewater SARS-CoV-2 surveillance for monitoring COVID-19 trends and early warning in Sweden, 2023–2024
Source: PLoS One. 2026 Sep 21;21(9):e0358326. doi: 10.1371/journal.pone.0358326 (PMC13592587; doi:10.1371/journal.pone.0358326)
Supplement: Supplementary file 2 [file pone.0358326.s002.pdf]

S2 File. WWTP summary table

This supporting material is supporting information alongside the article *“Evaluation of wastewater SARS-CoV-2 quantification as a surveillance indicator for COVID-19 in Sweden, 2023–2024”.*

S2 Table. Summary of wastewater treatment plants (WWTPs) included in the study.
Summary of the 19 wastewater treatment plants (WWTPs) included in the analyses, including region, population within each catchment area, total regional population, and the proportion of the regional population covered. The table also includes aggregated data for the Stockholm region (4 WWTPs) and the national level (19 WWTPs).

WWTP	Region	Population in catchment area	Regional population	Regional population (%)
Västerås	Västmanland	145 000	280 713	52
Uppsala	Uppsala	191 000	400 682	48
Göteborg	Västra Götaland	800 000	1 758 656	45
Örebro	Örebro	137 000	307 772	45
Östersund	Jämtland	54 000	132 670	41
Umeå	Västerbotten	109 000	276 295	39
Henriksdal	Stockholm	875 000	2 440 027	36
Linköping	Östergötland	154 000	471 912	33
Gävle	Gävleborg	89 000	287 334	31
Luleå	Norrbotten	70 000	249 177	28
Kalmar	Kalmar	66 000	247 711	27
Malmö	Skåne	356 000	1 414 324	25
Karlstad	Värmland	70 000	283 976	25
Käppala	Stockholm	500 000	2 440 027	20
Jönköping	Jönköping	71 000	369 113	19
Bromma	Stockholm	375 000	2 440 027	15
Grödinge	Stockholm	345 000	2 440 027	14
Helsingborg	Skåne	149 000	1 414 324	11
Östhammar	Uppsala	4 500	400 682	1
Stockholm (4 WWTPs)	Stockholm	2 095 000	2 440 027	86
National (19 WWTPs)	National	3 917 000	10 521 556	43
