# Supplementary material for: Evaluation of wastewater SARS-CoV-2 surveillance for monitoring COVID-19 trends and early warning in Sweden, 2023–2024
Source: PLoS One. 2026 Sep 21;21(9):e0358326. doi: 10.1371/journal.pone.0358326 (PMC13592587; doi:10.1371/journal.pone.0358326)

S4 File. Map.

This supporting material is supporting information alongside the article *"Evaluation of wastewater SARS-CoV-2 quantification as a surveillance indicator for COVID-19 in Sweden, 2023–2024"*.

S4 Fig. Geographic distribution of included regions in Sweden.
Map of Sweden showing the geographical location of the 14 regions included in the study, out of the country's 21 regions. Map created by the authors in R using Natural Earth map data.

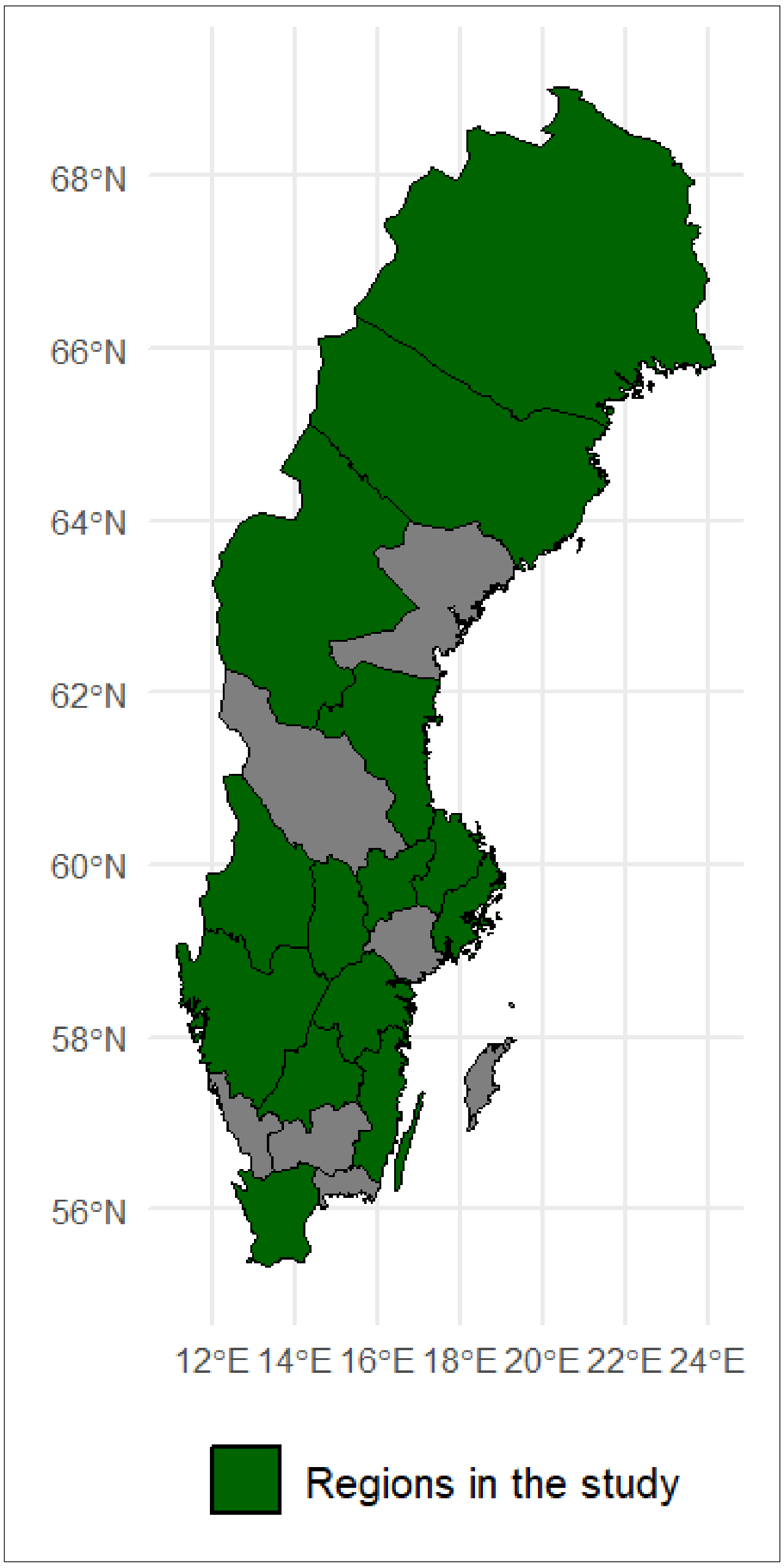

Supplement: Supplementary file 3 [file pone.0358326.s004.pdf]
