# Supplementary material for: Evaluation of wastewater SARS-CoV-2 surveillance for monitoring COVID-19 trends and early warning in Sweden, 2023–2024
Source: PLoS One. 2026 Sep 21;21(9):e0358326. doi: 10.1371/journal.pone.0358326 (PMC13592587; doi:10.1371/journal.pone.0358326)

S5 File. Cross-correlation results between wastewater SARS-CoV-2 levels and reported COVID-19 cases for weekly data, periods of increasing COVID-19 case numbers and periods of decreasing COVID-19 case numbers.

This supporting material is supporting information alongside the article *"Evaluation of wastewater SARS-CoV-2 quantification as a surveillance indicator for COVID-19 in Sweden, 2023–2024"*.

S2 Fig. Cross-correlation between wastewater SARS-CoV-2 levels and reported COVID-19 cases. Cross-correlation results for weekly data (72 weeks). Correlation coefficients (r) are shown for lags −3 to +3, where negative lags indicate that wastewater leads reported cases and positive lags indicate that wastewater lags reported cases. Results are presented for each of the 19 wastewater treatment plants (WWTPs), the Stockholm region (4 WWTPs), and the national level (19 WWTPs), together with the proportion of the regional population covered by each catchment area. The highest correlation coefficient for each WWTP is shown in bold.

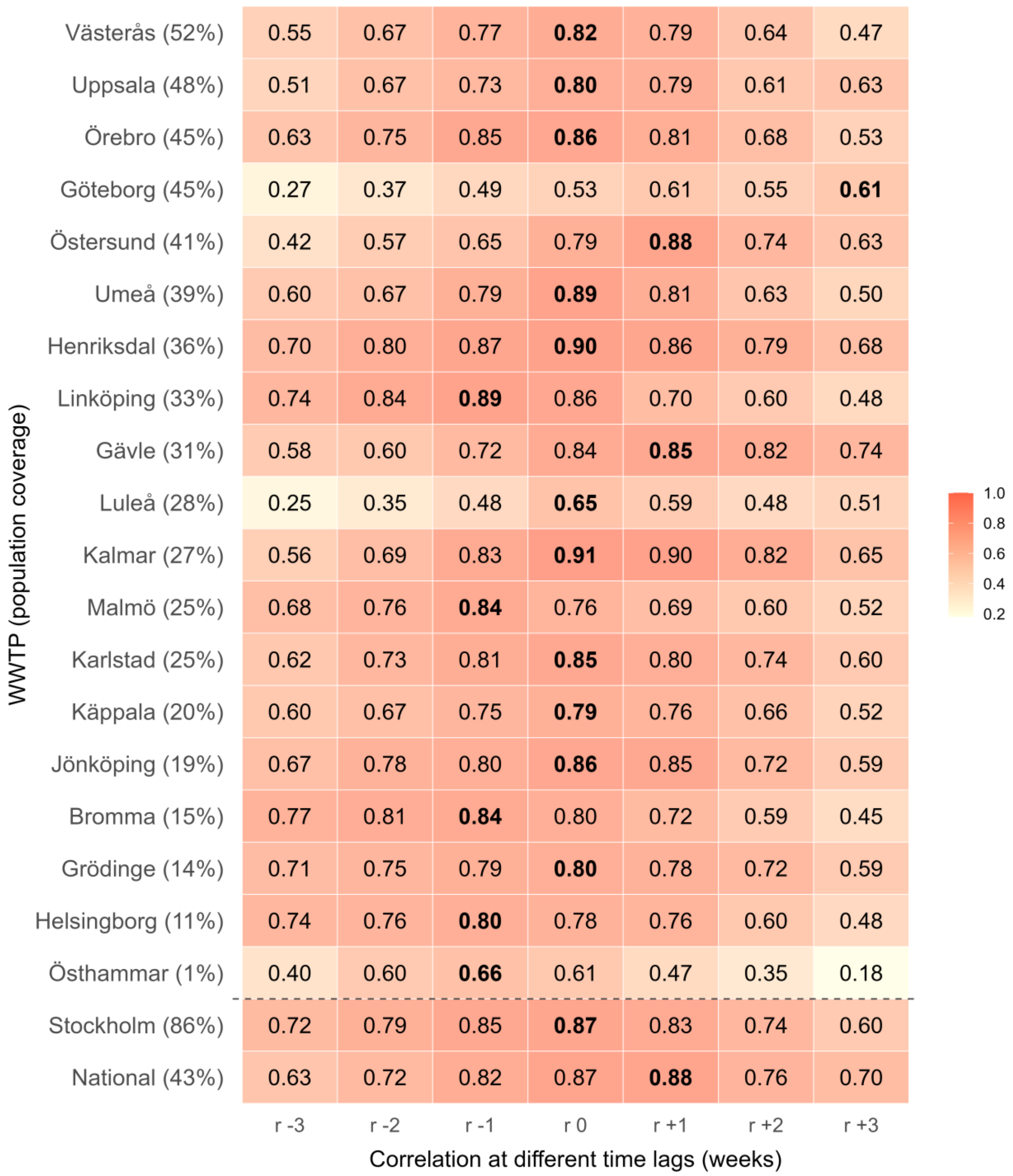

S3 Fig. Cross-correlation between wastewater SARS-CoV-2 levels and reported COVID-19 cases during weeks of increasing case numbers.

Cross-correlation results for 7 weeks of increasing case numbers. Correlation coefficients (r) are shown for lags −3 to +3, where negative lags indicate that wastewater leads reported cases and positive lags indicate that wastewater lags reported cases. Results are presented for each of the 19 wastewater treatment plants (WWTPs), the Stockholm region (4 WWTPs), and the national level (19 WWTPs), together with the proportion of the regional population covered by each catchment area. The highest correlation coefficient for each WWTP is shown in bold.

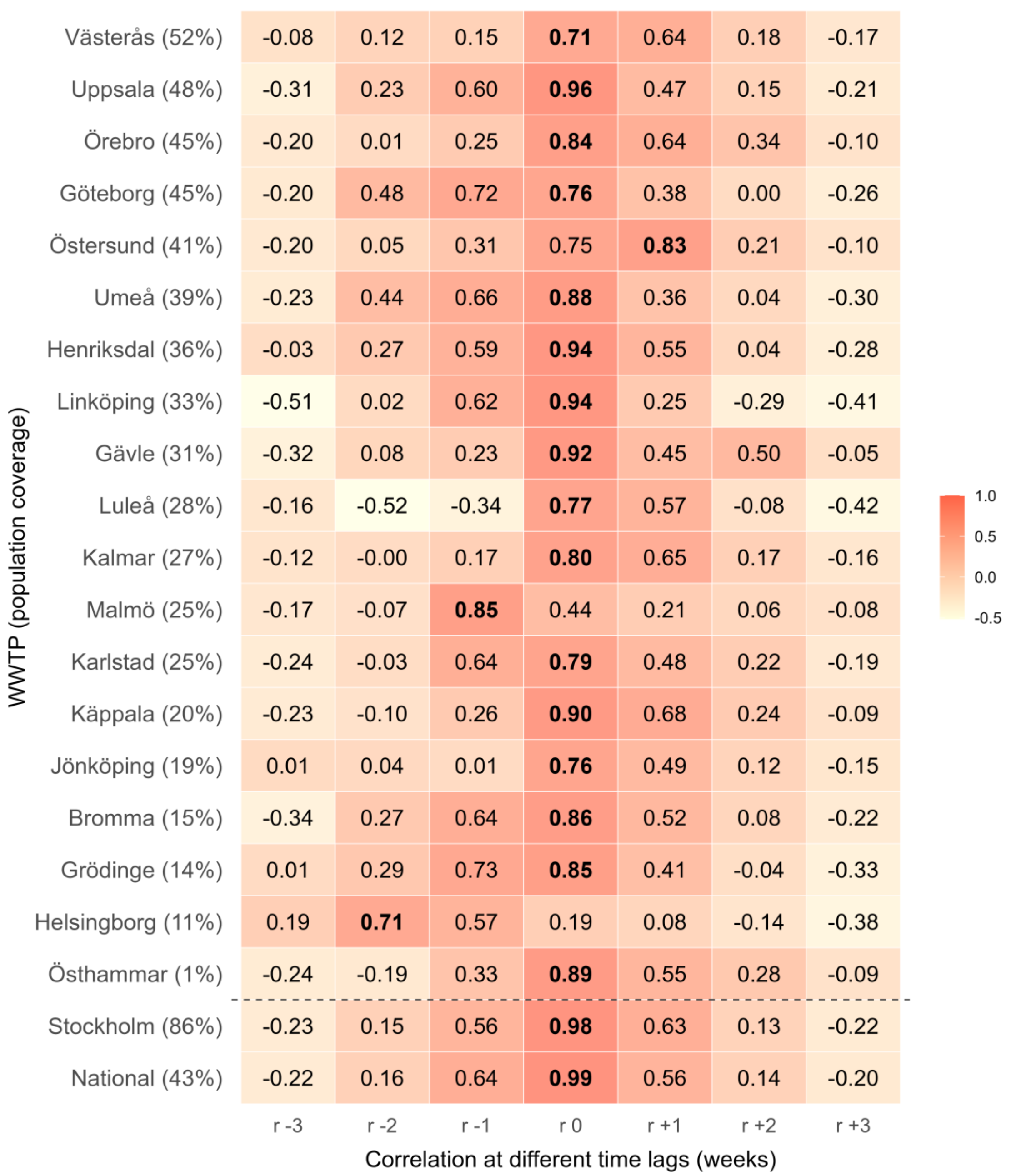

S4 Fig. Cross-correlation between wastewater SARS-CoV-2 levels and reported COVID-19 cases during weeks of decreasing case numbers.

Cross-correlation results for 7 weeks of decreasing case numbers. Correlation coefficients (r) are shown for lags −3 to +3, where negative lags indicate that wastewater leads reported cases and positive lags indicate that wastewater lags reported cases. Results are presented for each of the 19 wastewater treatment plants (WWTPs), the Stockholm region (4 WWTPs), and the national level (19 WWTPs), together with the proportion of the regional population covered by each catchment area. The highest correlation coefficient for each WWTP is shown in bold.

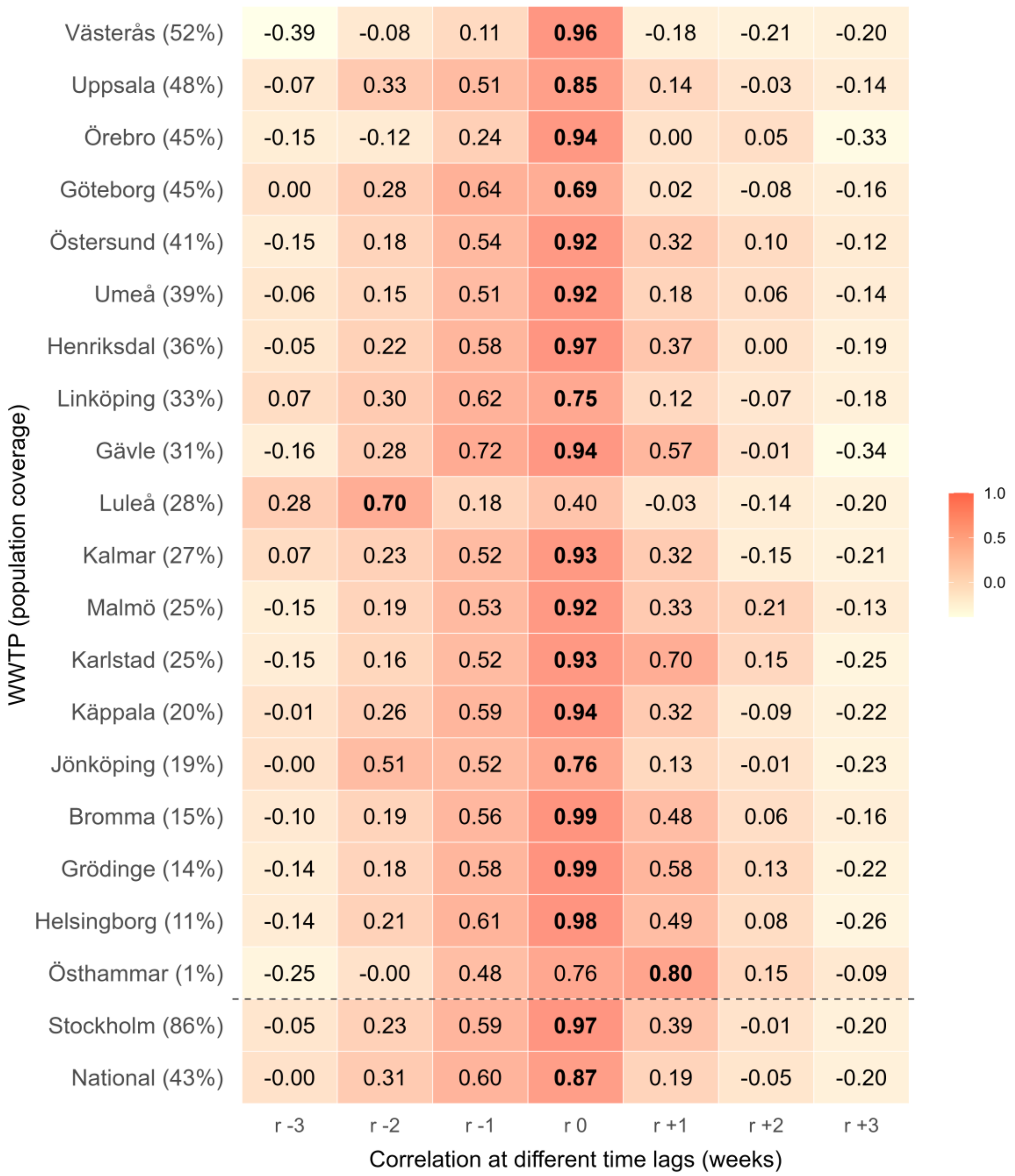

Supplement: Supplementary file 4 [file pone.0358326.s005.pdf]
