# Supplementary material for: Evaluation of wastewater SARS-CoV-2 surveillance for monitoring COVID-19 trends and early warning in Sweden, 2023–2024
Source: PLoS One. 2026 Sep 21;21(9):e0358326. doi: 10.1371/journal.pone.0358326 (PMC13592587; doi:10.1371/journal.pone.0358326)
Supplement: Supplementary file 5 [file pone.0358326.s003.docx]

# S3 File. Summary of the classification analysis.

This supporting material is supporting information alongside the article *“Evaluation of wastewater SARS-CoV-2 quantification as a surveillance indicator for COVID-19 in Sweden, 2023–2024”.*

**S2 Table. Summary of the classification analysis.**
Numbers of true positives (TP), false positives (FP), true negatives (TN), and false negatives (FN) underlying the performance metrics shown in Fig. 3 during the study period (July 2023–December 2024). Classification was based on wastewater SARS-CoV-2 levels compared with reported COVID-19 cases (reference surveillance indicator). Results are shown for two thresholds of increase (>10% and >25%) and for week-to-week changes and 3-week trends for the Stockholm and national levels. Weeks with wastewater SARS-CoV-2 levels below the 20th percentile were excluded from the analyses, as described in the Methods.

| **Analysis** | **Level** | **Change threshold** | **Weeks  included** | **TP** | **FP** | **TN** | **FN** |
| --- | --- | --- | --- | --- | --- | --- | --- |
| Week-to-week | Stockholm | >10% | 57 | 15 | 9 | 27 | 6 |
| Week-to-week | Stockholm | >25% | 57 | 5 | 9 | 36 | 7 |
| Week-to-week | National | >10% | 57 | 17 | 10 | 23 | 7 |
| Week-to-week | National | >25% | 57 | 8 | 10 | 33 | 6 |
| 3-week trend | Stockholm | >10% | 56 | 14 | 5 | 30 | 7 |
| 3-week trend | Stockholm | >25% | 56 | 7 | 6 | 41 | 2 |
| 3-week trend | National | >10% | 56 | 13 | 6 | 32 | 5 |
| 3-week trend | National | >25% | 56 | 7 | 7 | 38 | 4 |
